# Single Image Dehazing Algorithm Analysis with Hyperspectral Images in the Visible Range

**DOI:** 10.3390/s20226690

**Published:** 2020-11-23

**Authors:** Miguel Ángel Martínez-Domingo, Eva M. Valero, Juan L. Nieves, Pedro Jesús Molina-Fuentes, Javier Romero, Javier Hernández-Andrés

**Affiliations:** Department of Optics, Faculty of Science, University of Granada, 18071 Granada, Spain; martinezm@ugr.es (M.Á.M.-D.); jnieves@ugr.es (J.L.N.); pedromf@correo.ugr.es (P.J.M.-F.); jromero@ugr.es (J.R.); javierha@ugr.es (J.H.-A.)

**Keywords:** dehazing, defogging, spectral imaging, image quality, wavelength

## Abstract

In foggy or hazy conditions, images are degraded due to the scattering and attenuation of atmospheric particles, reducing the contrast and visibility and changing the color. This degradation depends on the distance, the density of the atmospheric particles and the wavelength. We have tested and applied five single image dehazing algorithms, originally developed to work on RGB images and not requiring user interaction and/or prior knowledge about the images, on a spectral hazy image database in the visible range. We have made the evaluation using two strategies: the first is based on the analysis of eleven state-of-the-art metrics and the second is two psychophysical experiments with 126 subjects. Our results suggest that the higher the wavelength within the visible range is, the higher the quality of the dehazed images. The quality increases for low haze/fog levels. The choice of the best performing algorithm depends on the criterion prioritized by the metric design strategy. The psychophysical experiment results show that the level of agreement between observers and metrics depends on the criterion set for the observers’ task.

## 1. Introduction

In foggy or hazy conditions, images are degraded due to the scattering and attenuation by the atmospheric particles, reducing the contrast and visibility, distorting the color and decreasing the visibility of the content in the scene and making the object features difficult to identify by humans and by computer vision systems. This degradation depends on the distance between the object and the acquisition device, the type (i.e., haze and fog water particles have different sizes) and the density of atmospheric particles and also on the wavelength. The techniques to eliminate this degradation, known as dehazing (or defogging), can improve the visual quality of images for computer-aided applications and human interpretation and are, consequently, crucial in many applications such as air and maritime transport, surveillance, driver assistance systems, remote sensing, agronomy, archaeology, astronomy, etc. [1,2,3,4,5,6,7].

Dehazing methods can be divided [2] into three categories: image enhancement [8,9,10], image fusion [11] and image restoration-based methods [12]. Another classification is made according to how many images are used: single image methods or multiple image methods [13]. Although many methods [14] have been proposed and compared, here, we focus on five well-known different single image dehazing color based methods which rely on different approaches. These five methods have been selected because they are quite recent and provide very good results according to some surveys [2,15], and also because they require neither user interaction nor additional information (such as depth maps, atmospheric parameters or some prior knowledge about the images), which is one of the requisites of real-world computer vision applications.

The performance of dehazing methods must be assessed and this is done by evaluating the quality of the recovered images, which can be done both subjectively and objectively. Whilst numerous different image quality metrics have been proposed, the evaluation of the quality requires more research [2,16]. Here, we will use eleven quality metrics representing different design strategies and that are commonly used in the dehazing domain.

To evaluate dehazing algorithms, it is also essential to have image databases that are sufficiently representative of different capture conditions and scene contents. Recently, technological advances in image sensors and spectral filtering have allowed the proliferation of multispectral and hyperspectral systems for image capture in a wide range of applications. However, despite their potential, they have not yet been used in dehazing methods. Recently, a spectral hazy image database for assessment (SHIA) has become available [17], which was inspired by a previous color image database (CHIC) from the same authors [18]. SHIA [17] includes a set of spectral images with various levels of haze/fog generated with a fog machine, with the corresponding fog-free spectral image which serves as the reference image (ground truth). The difference between haze and fog is based on the radius size of the water particles (less than 1 micron corresponds to haze) and the concentration (more than 100 particles/cm^3^ is considered as fog) [19]. Since no experimental data are provided on the particle concentration in this database, we cannot be sure about the haze/fog distinction, so we will use both terms throughout the text of this paper. The fog-free image allows the use of different quality metrics, including those that require a reference image for comparison.

Despite the fact that in many applications of dehazing algorithms the final decision has to be made by humans, very few studies have considered subjective evaluations of dehazing algorithms [16,20,21], although some authors have investigated how human observers are sensitive to changes in the naturalness of colors due to atmospheric effects [22]. In our study, we have run two psychophysical experiments where the subjects were asked to rank a set of images according to a given quality criterion.

This paper focuses on the use of dehazing methods with hyperspectral images in the visible spectrum, evaluating five state-of-the-art single image dehazing algorithms originally developed to work on color images by using eleven different image quality metrics and by running a psychophysical experiment with 126 subjects. Our main goal was to find answers to the following questions: are dehazing algorithms, originally developed to work on RGB images, able to deal with spectral images? Would they perform better for particular wavelengths? Is there any dependency between the performance of dehazing methods and fog densities? Do metrics for image quality assessment correlate with the perceptual judgment of human observers? El Khoury et al. [17] also tackled some of these questions but, here, we have used different dehazing algorithms, including the recent Berman method [23], a more comprehensive set of metrics, and we incorporate a psychophysical experiment that was not considered in the El Khoury et al. [17] paper.

The main contributions of this work are two: the first one is to use single image dehazing algorithms, originally developed to work on RGB images, on a spectral hazy image database in the visible range. The second one is that the evaluation has been done using both objective metrics and a psychophysical experiment in a more comprehensive way than in previous studies.

The paper is organized as follows: Section 2 reviews the five dehazing methods tested and the spectral hazy images utilized, describes the eleven image quality metrics used and presents the subjective evaluation experiments. Section 3 analyzes our results, and the conclusions and future work are given in Section 4.

## 2. Methods

### 2.1. Dehazing Algorithms

Methods for improving the visibility of images affected by haze or fog are classically classified into those that do not use physical models (using only the statistical information contained in the image) [8,9,10] and those that do use a physical model of atmospheric scattering [24] (and that explain the interaction of the atmospheric particles with the light that comes to us from objects present in a scene). We can also find an alternative classification (but included in the latter) which distinguishes between multiple image methods, polarizing filter-based methods, methods with known depth, single image methods and deep learning-based methods. During the last 20 years, numerous proposals have been made, and not always inspired by the physical phenomena underlying atmospheric optics that can limit the visibility of images. Two detailed reviews of all proposals up to 2016–2017 can be found in [2,15].

In general, any physical dehazing method describes the image radiance *I*(*x*) by combining the direct transmission and airlight terms as

(1) $$I\left(x\right)=L_{o}\left(x\right)e^{-\beta d\left(x\right)}+L_{\infty }\left(1-e^{-\beta \;d\left(x\right)}\right)$$

where *x* denotes the 2D pixel coordinates in the image, *L_o_* is the radiance coming from the spatial location *x* (i.e., the object), *β* is the atmospheric extinction coefficient, *d(x)* the distance between the object and the camera and $L_{\infty }$ is the horizon radiance; the airlight *A(x)* is usually defined as the term $A\left(x\right)=L_{\infty }\left(1-e^{-\beta d\left(x\right)}\right)$, and the transmission as $t\left(x\right)=e^{-\beta \;d\left(x\right)}$. Hence, the scattering model in the above equation which describes the image captured by the camera can be written simply as

(2) I(x)=D(x)+A(x)

where $D\left(x\right)=L_{o}\left(x\right)t\left(x\right)$ represents the so-called direct transmission.

In this study, we have used only five of the most representative dehazing methods, originally developed to work on RGB images. They have been adjusted to be applied on spectral images rather than color images, by building a monochrome three-channel image from a particular wavelength with the same information in every channel.

The five dehazing methods evaluated are named here as: dark channel prior method [25], Tarel method [26], Meng method [27], DehazeNet method [28] and Berman method [23]. The rest of this sub-section is dedicated to a brief description of these five algorithms.

#### 2.1.1. The Dark Channel Prior (DCP) Method

This well-known model [25] falls within the category of “single image” since the authors use a single channel as input information. The algorithm is based on the assumption that for a given pixel in a color RGB image, one channel (R, G or B) has low intensity at some pixels (this constraint is supported by the statistics of natural scenes where there is a multitude of shadows and highlights). This channel is the so-called dark channel prior $\tilde{t}\left(x\right)\;$and an estimate of the airlight can be obtained from it as

(3) $$\tilde{t}\left(x\right)=1-\underset{y\in \Omega (x)}{\min}\left(\underset{c}{\min}\frac{I^{c}\left(x\right)}{{L_{\infty }}^{c}}\right)$$

where *I^c^(x)* stands for the image *I*(*x*) recorded in the channel c (i.e., R, G and B, or any other spectral channel), and ${L_{\infty }}^{c}$ is obtained by picking the top 0.1% brightest pixels in the dark channel. From there, the equation of the dichromatic model of atmospheric scattering is solved to obtain the recovered image. Since the algorithm is applied to patches *Ω(x)* in the scene, the model also includes a smoothing step to avoid pixelated results in the recovery image.

#### 2.1.2. The Tarel Method

This method [26] is a single image filtering-based method that imposes the initial condition that the fog corresponds to the most achromatic area (pure white) of the scene. Thus, the model imposes a white balance of the image and establishes that the airlight *A(x)* must be less than the smallest value of the image intensity *Ic(x)* in each channel c after normalization. The objective of the algorithm is to find the optimal value of *A(x)* that satisfies the previous condition, which is computationally expensive and requires additional heuristic rules to find it. In addition, the rules applied to find this optimal value may cause the model to have problems with scenes in which the color varies considerably between the objects in the scene, since the colors in the recovered image will tend not to correspond to the original. However, since the images used in this study are spectral images and not properly RGB images, this will not be a serious problem.

#### 2.1.3. The Meng Method

This method [27] extends the use of the idea of the dark channel prior to estimate transmission *t*(*x*) by imposing lower bounds on this transmission for its search. Starting from the image formation equation, it models the transmission as

(4) $$t\left(x\right)=\frac{A^{c}-I^{c}\left(x\right)}{A^{c}-C_{0}^{c}}$$

where $C_{0}^{c}$ is a scalar, so that the transmission values of the complete image are obtained by regularization of this expression for two scales $C_{0}^{c}$.

#### 2.1.4. The DehazeNet Method

This method [28] is a machine learning approach for single image haze removal. It uses a convolutional neural network (CNN) for the “learning” of the transmission function *t*(*x*). The CNN network, called DehazeNet in this study, is made up of four sequential feature extraction operations, multi-scale mapping, local extremity and non-linear regression and three layers of convolution. The authors used a training set of thousands of hazy image patches that were synthesized from haze-free images using a haze model with random *t*(*x*) transmission and assuming a white term for the light from the atmosphere L_∞_.

#### 2.1.5. The Berman Method

The model [23] proposes a prior non-local haze and assumes that the colors that make up the fog-free scene can be described by a finite number of colors (or clusters in the RGB gamut of the imageTherefore, the effect of the fog will be a linear elongation or extension (haze-line) of these reference clusters according to different transmission coefficients as a function of the distances of the objects to the camera. The transmission function is calculated from different haze-lines and the final transmission map is obtained after a smoothing step.

### 2.2. Spectral Image Database

We use, here, images from the spectral hazy image database for assessment (SHIA) [17]. SHIA consists of two real indoor scenes, M1 and M2, under ten different levels of fog each (level 0 corresponds to the maximum level of fog, whereas level 9 to the minimum), generated with a fog machine, and their corresponding fog-free (ground truth) image, captured in the visible and the near infrared (450 to 1000 nm with a spectral resolution of 10 nm). According to El Khoury et al. [17], the generated fog in the experiment, composed of water droplets, had radius values similar to real atmospheric fog.

El Khoury et al. [17] used two different liquid crystal tunable filters (LCTFs): one to achieve images from 450 to 720 nm and the other to achieve images from 730 to 1000 nm. The integration time was fixed to 530 ms for all the images and wavelengths. The fact that the integration time is the same for all wavelengths increases the noise for low wavelengths due to the small irradiance impinging on the sensor caused by the low CMOS responsivity and the low LCTF transmittance at wavelengths below 500 nm.

All the images from the SHIA database have the same size of 1312 × 1082 pixels. The dataset used here consists of only the M1 scene that was acquired over the visible range (450–720 nm, every 10 nm) under 10 different levels of fog besides the fog-free scene. The authors of SHIA [17] carried out a dark correction and a spectral calibration for all the images.

**Figure 1:**
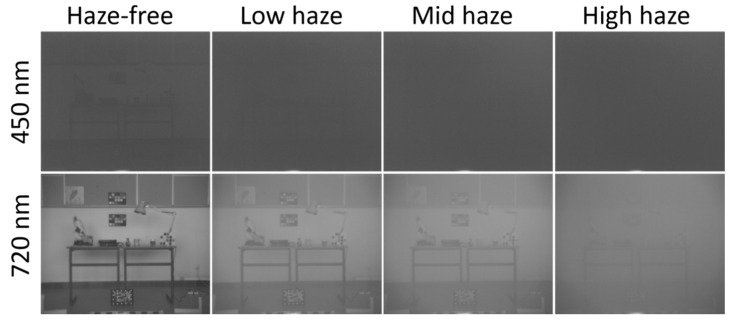
Example images from the spectral hazy image database for assessment (SHIA) [17], at two different wavelengths and four different levels of haze.

From the 10 levels of fog measured by [17], in this analysis we have selected only three levels of fog (level 9 named as “low”, level 7 as “medium” and level 5 as “high”) because for levels 0 to 4 (very dense fog), there is almost no information on the spectral images. Figure 1 shows some examples of these images.

### 2.3. Image Quality Metrics

To evaluate the perceptual quality of a dehazing method applied to images degraded by haze or fog is not a minor question, and currently there is no consensus on which metric or metrics should be used and which one best correlates with human judgments [2,18].

The objective assessment of the different dehazing methods evaluates quantitatively the image according to some features. Many different metrics are used in the literature of dehazing algorithms [2] and they can be classified in three main categories depending on the availability of a reference image: full-reference (the reference is the fog-free image), reduced-reference (the reference is the foggy or hazy image) and no-reference metrics [18]. For this research, we have a reference image which is the fog-free image described in Section 2.2 and therefore we can test any proposed metric from the three categories.

We have made an initial selection of eleven metrics (described below) which are quite commonly used for the quality assessment of dehazed images. Five of them are no-reference metrics, four are full-reference metrics and two are reduced-reference metrics.

In Section 3.1, we analyze the influence of several factors on image quality. Due to space issues, we have selected a sub-group of six metrics which are representative of different design strategies: detecting new edges in the dehazed image compared to the hazy image (e descriptor); detecting changes in the first or second derivatives in the dehazed image (GMG descriptor); detecting contrast enhancement (Std metric); assessing similarity between the dehazed and haze-free images (PSNR); assessing the amount of information contained in the dehazed image (entropy); trying to model human visual system quality assessment (SSIM). In Section 3.2, we will use the full set of eleven metrics to evaluate the level of agreement with the observers’ judgements obtained from the psychophysical experiments.

In addition to the designed strategy, we have also considered the level of agreement with the observers’ evaluation of image quality as a second criterion for choosing the subset of relevant metrics. If a given design strategy is present in more than one metric, we selected as representative of that particular design strategy the metric which showed a higher level of agreement with the observers’ answers to the first question of our psychophysical experiment.

#### 2.3.1. e Descriptor

This metric accounts for the amount of new visible edges after dehazing [29,30]. This is calculated using the number of edges in the restored image, and the number of edges in the hazy image. The hazy image is then the reference image, since the haze tends to fade edges. Hence, a restored image with a higher number of edges (i.e., with a higher value of the e descriptor) is considered as a better-quality restored image. However, care must be taken when dealing with this metric, since high-frequency spurious noise, such as salt and pepper noise (which might be introduced by the dehazing algorithms), could introduce new false edges and thus increase the value of the e descriptor. This could also happen in the hazy image if it is noisy and false edges are detected. This issue can be partially accounted for by refining the edge detection stage of the process for both the hazy and the restored image.

#### 2.3.2. r Descriptor

This metric is the geometric mean of the visibility level [29,30]. It is calculated using the set of visible edges in the restored image, and the ratio between the gradient at each visible edge of the restored image over the gradient of the same point on the original hazy image. This descriptor is complementary to the e descriptor. It yields a higher value the greater the amount of edges restored in the dehazed image is. The same issue regarding the detection of false edges could arise using this metric in the restored or hazy images.

#### 2.3.3. σ Descriptor

This is the normalized number of lost pixels [29,30]. In this context, a pixel is considered to be a lost pixel if it was properly exposed in the reference image (usually the hazy image), but it becomes saturated or underexposed in the restored image. A pixel is considered to be saturated if it is above 98%, and underexposed if it is below 2% of the dynamic range. This metric is calculated using the number of new lost pixels in the restored image and the total number of pixels in the image. This metric belongs to the category of full-reference methods. Its design strategy is markedly different from the other selected metrics, and it is expected to correlate poorly with the observers’ quality judgments, unless the amount of pixel loss is really high. This is never the case for the SHIA image database. Residual fog or haze do not necessarily result in saturated or underexposed pixels.

#### 2.3.4. Laplacian Descriptor (LAP)

This metric is calculated by convolving a 3 × 3 Laplacian kernel across the entire image and calculating its mean value [30,31]. In a completely homogeneous image, LAP would equal zero. The less homogeneous the image is, the higher the value of LAP is. Heterogeneity has to do with the presence of edges or fine detail, so we could expect an increase in the LAP value from a hazy image to a restored image. This metric uses a design strategy based on computing second derivatives of the dehazed image, and it is a non-reference metric.

#### 2.3.5. Gray Mean Gradient (GMG)

This metric is related to the texture characteristics of the image, especially its edge information [30,32]. The higher the value of GMG, the more visible the edges are in the image. Thus, this metric is also supposed to increase its value when comparing the hazy image with the restored image. It is a non-reference metric.

#### 2.3.6. Standard Deviation (Std)

This metric is related to the heterogeneity of the distribution of the gray levels in the image. In a completely homogeneous image, Std would equal zero. Ideally, hazy images are far more homogeneous than non-hazy ones. Hence, a restored image is expected to yield a higher Std value compared to the hazy one. Care must be taken here since the presence of noise could also increase the value of *STD* even if the objects are not more visible in the restored image. *STD* is computed as shown in Equation (5):

(5) $$STD=\overset{U}{\underset{i=1}{\sum }}\overset{V}{\underset{j=1}{\sum }}\sqrt{\left(\frac{{\left(g\left(i,j\right)-\mu \right)}^{2}}{N}\right)}$$

where *μ* is the mean gray value of the image, *g*(*i*,*j*) is the gray value present in pixel whose spatial coordinates are *i*, *j* and *N* is the number of pixels in the image. It is a non-reference metric.

#### 2.3.7. Information Entropy

According to information theory, if an image is considered as a source of information, the larger the value of entropy is, the greater the amount of information the image contains [2]. It is calculated taking into account all possible gray levels and their probability of happening in the image (its frequency in the normalized image histogram). If a dehazing method restores information that was lost due to the haze in the hazy image, the entropy should increase from the hazy image to the restored image. It belongs to the category of non-reference metrics.

#### 2.3.8. Mean Square Error (MSE)

This metric directly compares two images [2]. It is the mean of the squared differences between the two images. This metric makes sense if the non-hazy reference image is available. Since we could expect any dehazing method to recover a restored image as close as possible to the non-hazy image, then a low MSE value would be desirable when comparing restored and ground truth non-hazy images. If we compare two identical images, the MSE value would be zero. This metric is sometimes misinterpreted as being proportional to the presence of noise in the restored image. However, differences between restored and ground truth images do not necessarily correspond with the presence of noise. MSE is sensitive to offsets in the mean gray level and contrast increments, amongst others. It is a full-reference metric belonging to the similarity assessment design strategy.

#### 2.3.9. Peak Signal to Noise Ratio (PSNR)

This metric is proportional to MSE, and therefore yields the same information [2]. It is computed as shown in Equation (6):

(6) $$PSNR=10\cdot \log\frac{f_{max}^{2}}{MSE}$$

where *f_max_* is the largest possible gray value. It is also a similarity assessment metric. In this case, the higher the value of *PSNR*, the more similar two images are. Again, it makes sense to use this metric for comparison between the restored image and the ground truth non-hazy one. Despite its name, the use of this metric in this context does not necessarily have to do with noise. A low *PSNR* could be due to mean gray level or contrast differences. It belongs to the group of full-reference metrics.

#### 2.3.10. Structural Similarity Index Measure (SSIM)

This metric was proposed in order to assess image quality from a perceptual point of view, taking into account that human visual perception is highly adapted to extracting structural information from a scene [33]. Three comparison functions (comparing luminance l, contrast c and structure s) are defined using the mean and standard deviation of the two images to be compared. These three functions are then combined as shown in Equation (7):

(7) $$SSIM=l{\left(i,j\right)}^{\alpha }\cdot c{\left(i,j\right)}^{\beta }\cdot s{\left(i,j\right)}^{\gamma }$$

where *α*, *β* and *γ* are positive-valued parameters to adjust the relative importance of each (usually set to 1). The closer to the value of *SSIM* to 1, the more similar the compared images are. It is then a full-reference metric within the group of perceptual-based design strategies.

#### 2.3.11. Natural Image Quality Evaluator (NIQE)

This blind image quality assessment metric is based on the construction of a collection of statistical features based on a space domain natural scene statistic model [34,35]. It does not need any a priori knowledge to be applied (non-reference metric). It evaluates the distance between the model statistics and the analyzed image statistics, which is proportional to the human judgment on the naturalness of an image.

In Table 1, we show a summary of the information contained in this sub-section relative to the classification of the different metrics used.

### 2.4. Subjective Evaluation

In addition to the objective assessment through different metrics previously described, two psychophysical experiments designed as a survey have been run, with 126 participants.

In this psychophysical experiment, we gave clear instructions to the participants so that they could carry it out from their homes on their personal computers. The age range of the observers was 19 to 65 years (mean = 40), of which 51.5% were female and 48.5% were male. All subjects gave their informed consent for inclusion before they participated in the study. The study was conducted in accordance with the Declaration of Helsinki, and the protocol was approved by the Ethics Committee of the University of Granada (1748/CEIH/2020).

The survey included two questions: the first one asked the observers to judge the similarity between the haze-free and dehazed images, and the second asked the observers to judge the increase in visibility for the objects present in the scene after dehazing without taking into account the similarity to the original haze-free image. These two aims are tackled by running two different psychophysical scaling experiments. For the first one, we used a rank order method and, for the second one, we used a preference selection method.

For the first question, using a rank order method, the subjects were presented with six images (the haze-free image and five dehazed images obtained with the five methods presented in Section 2.1) and they were asked to rank them according to a criterion of similarity with the haze-free image. Participants were asked to assign a rank from 5 (most similar) to 1 (least similar) according to the similarity between the dehazed and haze-free original images. They were not allowed to assign the same rank to more than one image. The images were randomly placed on the screen for each participant, in order to avoid any selection bias based on location.

For the second evaluation using a preference selection method, the participants were asked to only select one of the five algorithms which provided the highest visibility in the image after dehazing. There was no reference image to compare with in this second experiment. Five images were simultaneously presented (one per dehazing method) and only one image had to be selected by the participants. In this experiment, images were also randomly placed on the screen for each experiment in order to avoid any location bias.

For both psychophysical experiments, the observers were presented with the images corresponding to the five dehazing methods, three different levels of fog (low, medium and high) and three representative wavelengths (530, 620 and 710 nm), except for the highest fog condition, for which only two wavelengths were presented (620 and 710 nm) because at 530 nm, the images had almost no information. All the images presented to the observers were gray images since we are dealing with particular bands of spectral images.

The group of 126 observers included both people familiar with image quality features and naive observers. Although results can be biased by the previous knowledge and experience of the observers, and also by their emotions, motivation and other factors [2], we believe that for some applications in which the final decision, based on the image quality, is made by humans, this survey is very valuable. It allows us to make comparisons with objective evaluations carried out using different metrics, as explained in Section 3.

## 3. Results

**Figure 2:**
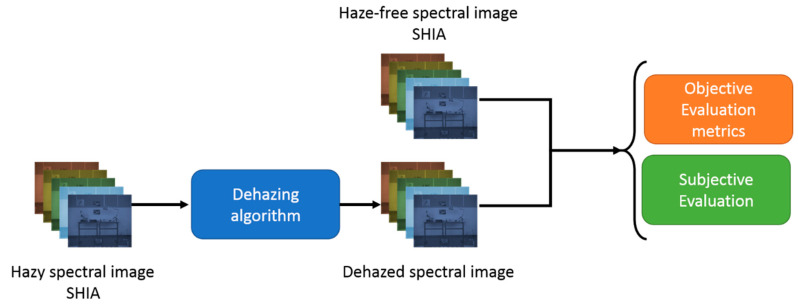
Workflow for the objective and subjective evaluation.

In this section, we first perform an objective analysis based on the image quality metrics values (Section 3.1). We analyze the influence of wavelength in both hazy and dehazed images, and also the influence of haze/fog level and the differences found among dehazing algorithms. Then, we analyze the results of the psychophysical experiments (Section 3.2), also assessing the level of agreement between metric values and observers’ judgements. Figure 2 shows the workflow followed in this work.

### 3.1. Evaluation Through Image Quality Metrics

#### 3.1.1. Influence of the Spectral Band on Hazy and Dehazed Images

**Figure 3:**
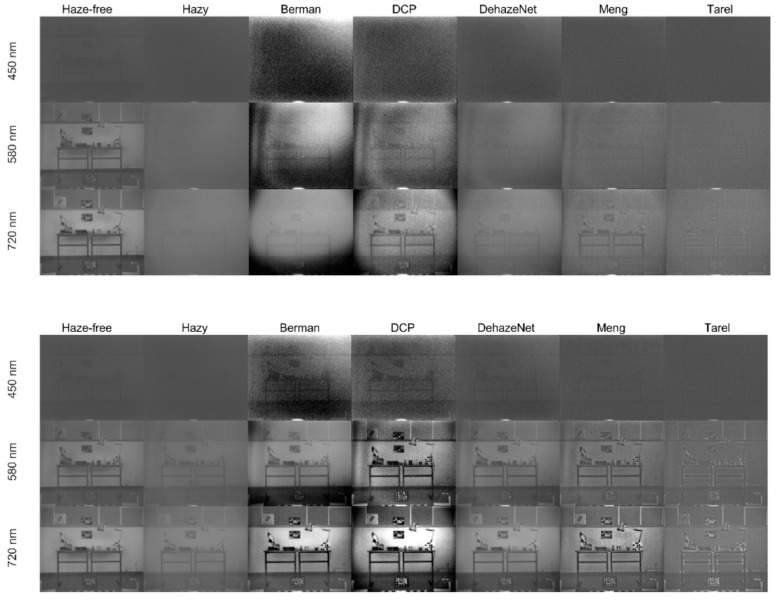
Original haze-free (first column), hazy (second column) and dehazed (columns 3 to 7) images for wavelength 450 nm (first row), 580 nm (second row) and 720 nm (third row). The images correspond to low haze conditions (upper figure) and high haze conditions (lower figure).

In Figure 3 (first two columns), we show the original haze-free and hazy images from the database at three wavelengths, for low and high haze levels. The image quality for the low wavelength range (up to 550 nm) is noticeably worse than for the higher wavelengths. This can be explained by considering two factors: the first is that the images have been captured with the same exposure for all wavelengths, leading to a small amount of irradiance impinging on the sensor for the short wavelength range, because of the low LCTF transmittance and sensor responsivity in this spectral range [17], as mentioned in Section 2.2. The second is that for hazy images, the attenuation is higher in this spectral range, causing an additional decrease in quality. In view of this circumstance, we have decided to consider only the wavelengths above 550 nm for detailed analysis, although we will also comment on the results obtained for the low wavelength range in this section.

**Figure 4:**
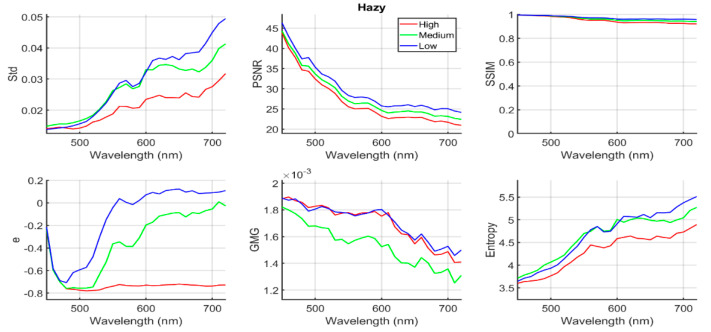
Quality metric values for the hazy images corresponding to low (blue), medium (green) and high (red) haze conditions, in the full wavelength range from 450 nm to 720 nm.

In Figure 4, we represent the six quality metrics considered (Std, PSNR, SSIM, e, GMG and entropy, as described in Section 2.3) for the hazy images (before applying the dehazing algorithms).

For the PSNR, SSIM and e metrics, we have taken as reference the haze-free image. The trend observed in Figure 4 is that the contrast (as measured by the Std metric) increases as the wavelength increases for the three haze conditions. This increase can also be related to the higher values of mean irradiance in the images as the wavelength increases, because the Std is not independent of signal level in the image. The values for the e metric are negative, which makes sense because we have taken the haze-free image as reference, which has a larger number of edges than any of the hazy images. The difference between hazy and haze-free images increases with the level of haze, as expected. The e results for the wavelength below 500 nm are the same for all three haze conditions, which is due to the low image quality of the images captured in this range. The e values tend to increase with wavelength for the medium and low haze conditions, reflecting that the higher the wavelength, the greater the number of visible edges. This is due to the increase in quality of the images and the decreasing effect of the haze, which results in a higher similarity between haze-free and hazy images. The positive e values for the low haze condition from 600 nm on might be caused by the fact that hazy and haze-free images were captured at different times, or by oscillations inherent to the edge counting method used by the metric.

The similarity between haze-free and hazy images decreases with wavelength, according to the PSNR and SSIM results. We can interpret these results considering that the image quality for the low-wavelength range is so low that adding haze to the image does not introduce very noticeable changes (see Figure 3, first row and first two columns). When the wavelength increases, the quality of the original images also increases, and thus the effect of haze becomes noticeable. As the wavelength increases, the effect of the haze also produces a change in mean intensity in the image, increasing the MSE value and contributing to the decrease in PSNR, with a higher relative decrease for the higher haze conditions.

**Figure 5:**
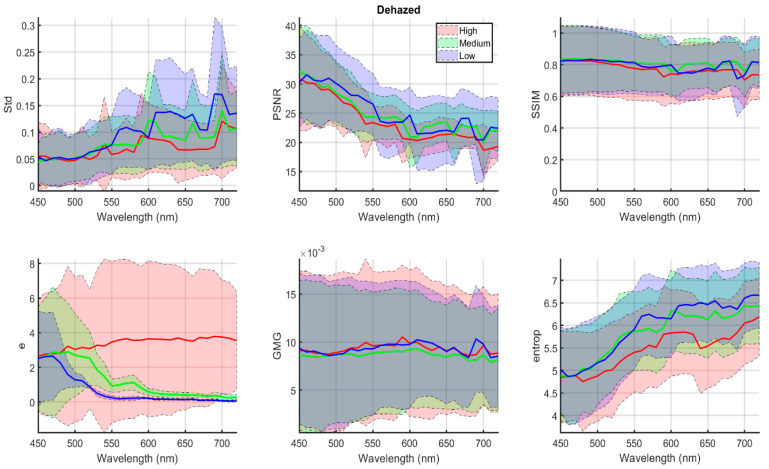
Quality metric values for the dehazed images (average of the five algorithms used for dehazing) corresponding to low (blue), medium (green) and high (red) haze conditions, in the full wavelength range from 450 nm to 720 nm. The shadowed areas correspond to plus/minus one standard deviation around the average results for each metric.

In Figure 5, we represent analogous data to Figure 4, but using the average across all methods of the quality metric results obtained for the dehazed images after using the five different algorithms applied for the dehazing. The shadowed areas correspond to plus/minus one standard deviation around the average results for each metric.

Again, for PSNR and SSIM images, we have taken as reference the haze-free image, but for the e metric, the reference was the hazy image, since after dehazing we wanted to see if the number of edges had increased, taking as a starting point the initial condition (hazy image).

In general, for all six metrics, we can find similar trends in the behavior of the metrics when the wavelength changes for the dehazed images rather than for the original hazy images. There are some variations depending on the haze condition and the algorithm applied to obtain the dehazed image, and the curves are rather spikier (a consequence of averaging five different dehazed images for each wavelength). For most metrics, we can also observe that the shadowed areas are rather wide, due to the high standard deviation values. This indicates that the variability in a given metric for different dehazing algorithms is high.

The Std values are higher for the dehazed images than for the hazy images, so we can conclude that the dehazing tends to increase the contrast in the image, as expected. Overall, the Std values tend to increase with wavelength, due to the better quality of the images in the long wavelength spectral range that we have commented on before. A better quality of the input image also results in higher contrast in the dehazed image.

The e metric values are positive for all wavelengths, as expected. The difference between the dehazed and hazy images is lower for the medium and low haze conditions than for the high haze condition. This reflects the fact that the dehazing algorithms introduce more radical changes in the images due to the greater difficulty of dealing with the condition of high haze. The e metric behaves differently with wavelength for the medium and low haze levels than for the high haze condition, tending to increase in the latter case and decrease in the former. This might be a consequence of the images at higher wavelengths being less affected by haze (as commented before). If they are less affected by haze, then the dehazing algorithms can work slightly better to improve the number of edges in the dehazed images, and this is especially noticeable in the high haze condition. For the medium and low haze conditions, the effect of the haze itself is less noticeable, and so the difference between hazy and dehazed images is also less noticeable as the wavelength increases.

The PSNR values in the low–medium spectral range tend to be lower for the dehazed images than for the hazy images. This reflects the fact that some dehazing algorithms tend to produce a quite high increase in contrast and significantly alter the appearance of the scene (see, for instance, DCP and Berman dehazed images, in Figure 3 columns 3 and 4). Thus, these algorithms produce images that have a less hazy appearance, but they are also less similar to the original image. This fact has also been found in a previous study using the same database [17].

The SSIM average results tend to present some oscillations as well, although the values for the high haze condition are lower than for the medium–low haze levels. The separation between medium and low levels is less noticeable than for the other metrics. This is a consequence of variable results as well the different algorithms applied (in particular, Berman is much more prone to oscillation in the quality metric results). In general, the SSIM values for dehazed images are lower than for the hazy images, which we also interpret in terms of the changes in contrast and general appearance of the images introduced by the dehazing process in most of the algorithms, in agreement with the results found by [17].

The GMG values for the medium haze levels are lower than for the other haze conditions, consistent with the results for the hazy images (see Figure 4). The oscillations in value make it somewhat difficult to interpret the trend with wavelength, but it seems that the higher GMG values correspond to the middle wavelengths. This does not necessarily indicate better quality overall of the dehazed images in this range (at least according to the rest of the metric results) and might suggest that GMG is more sensitive to artifacts introduced by the dehazing algorithms than the other metrics.

The entropy values increase with wavelength (as was also found for the hazy images), and they are consistent with worse quality of the dehazed images for the high haze level, in agreement with most other metrics and showing the expected behavior. The entropy is higher for the dehazed images than for the hazy images, due to the increase in contrast introduced by most dehazing algorithms.

Due to the inherent variability in the data, which can be deduced from the width of the shadowed areas in Figure 5, we have performed a statistical analysis to test if the trends observed are significant. The test is a one-way ANOVA with factor “wavelength”, performed independently for each haze level. For the low and middle haze levels, we found that the differences in metric values for different wavelengths are significant for PSNR, e and entropy (with *p* values between 1.38 × 10^−8^ and 0.031; F values between 1.69 and 4.4). For the high haze level, the effect is only significant for PSNR (*p* = 2.01 × 10^−6^, F = 3.47).

To sum up, we have found that there is a trend to increased quality when wavelength increases, both for hazy and dehazed images. However, the changes in image contrast and appearance introduced by the dehazing procedures are so evident as to make the dehazed images less similar to the original image than the hazy images, as was also concluded in [17] in a less comprehensive analysis with three wavelengths.

#### 3.1.2. Influence of the Haze Level on Dehazed Images

In this section, we will restrict our analysis to the wavelength range above 550 nm, to avoid any influence of the lack of quality in the captured images that we have mentioned before.

Some inferences can be made regarding the effect of the haze level (condition) by looking at Figure 4 and Figure 5. For wavelengths above 550 nm and hazy images (see Figure 4), we see how all the metrics (except for GMG) tend to be ranked by the amount of haze present in the images, with higher quality for the low haze condition. This means that the lower the haze level, the greater the contrast in the images and also the higher the similarity to the haze-free image, which is as expected. For the dehazed images (see Figure 5), the metrics behave similarly for the hazy images regarding the effect of the haze level. These trends are, however, more difficult to extract from the previous figures because they have been designed to show the behavior with varying wavelengths.

**Figure 6:**
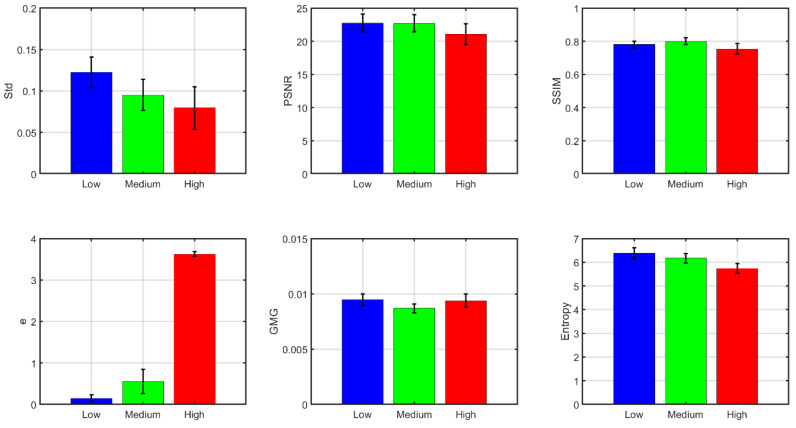
Quality metrics for the dehazed images (average of all wavelengths above 550 nm and the five algorithms tested). Standard deviations are indicated as error bars in the figure.

We then decided to look at the trends for the dehazed images by averaging results for all wavelengths above 550 nm and all the algorithms used (see Figure 6).

For all the metrics analyzed (except for GMG), the results are consistent with a decrease in image contrast or in similarity with the haze-free image (or hazy image for the e metric) when the level of haze increases. This means that, on average, the algorithms perform worse as the haze level increases. For the e metric, the increase in the number of edges present is probably a result of the dehazing algorithms introducing some spurious edges, such as artifacts for the high haze condition. The overall effect of haze level in the GMG values is very slight, showing that the unexpected behavior for the medium haze condition might not be very significant. There are some metrics which show higher sensitivity to the increase in haze, such as Std and e; for others, such as SSIM or GMG, the variation is less relevant. Some of the algorithms are less well equipped to deal with high haze levels than others, as will be described in the next sub-section. This tends to flatten the averaged metric values for the different conditions in some cases.

We have also performed a one-way ANOVA test with factor “haze level” using the data in Figure 6. The results show that the effect of haze level is significant for all six metrics, with a maximum *p* value of 4.45 × 10^−5^, and a minimum F value of 12.27 (corresponding to the GMG metric). We can see how the standard deviations in Figure 6 are considerably lower than in Figure 5. Figure 6 was obtained by averaging the results of the five different algorithms before performing the average across wavelengths and the ANOVA analysis. We can then conclude that the main source of variability for our data are the different levels of quality offered by the five algorithms tested.

#### 3.1.3. Comparison between the Algorithms Tested

**Figure 7:**
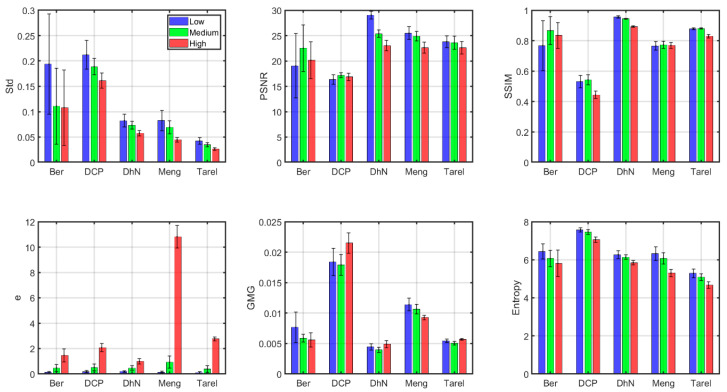
Quality metrics for the dehazed images (average of all wavelengths above 550 nm). Standard deviations are indicated as error bars in the figure.

In Figure 7, we present the average quality metric values across wavelengths above 550 nm separately for each of the five algorithms tested and each of the three haze conditions.

According to the image quality metrics, most algorithms achieve consistent results across wavelengths and for different haze levels, since they present the same trends in metric values for different haze conditions (quality ranked from the worst values in high haze conditions to the best values in low haze conditions). There are some exceptions to this rule, especially for Berman and DCP in the medium haze level. As shown in the previous sub-section, the Std and e metrics are more sensitive to haze conditions in general for all the algorithms. The data for the e metric indicate a larger number of edges introduced by the dehazing in the high haze condition, but according to the other metrics, the image quality of the dehazed image is consistently worse in the high haze condition (as expected). This might indicate that the borders introduced are mostly due to artifacts.

DCP produces some artifacts, such as vignetting in the outer areas of the images, as can be seen in Figure 3. DehazeNet produces more visually pleasing results, especially for the lowest haze condition, whilst Meng is consistent across wavelengths and haze levels, but less able to effectively decrease the loss of contrast in the images. The worst results are produced by Tarel in Std and entropy metrics, and DCP in PSNR and SSIM metrics. In general, the Tarel results are quite close to the Std values obtained by the original hazy images. This suggests that Tarel is not able to produce a very noticeable effect in transforming the appearance of the hazy original images. DehazeNet produces the worst result in e and GMG metrics, indicating that there are less spurious borders introduced in the dehazing process, and this algorithm tends to produce images similar to the haze-free image according to the PSNR and SSIM values.

Berman and DCP produce alterations in the naturalness of the images, increasing the contrast way above the original haze-free image. Whilst the metrics that are sensitive to contrast enhancement (Std, GMG, entropy) show high levels for these two algorithms (DCP especially), the metrics that are sensitive to similarity with the haze-free image produce worse results for Berman and DCP.

We have performed a two-way ANOVA test with all data used in Figure 7 (before averaging). We have tested the factors “algorithm” and “haze level” and their interaction was significant. We found that both factors and the interaction were significant for the six metrics analyzed, with the interaction being the least significant (minimum F value of 3.43, maximum p value of 0.00091 for the Std metric). We can then be confident in the trends found and commented on before regarding the different levels of quality obtained for each algorithm, the variations found according to haze level and the fact that some algorithms are more stable than others for different haze levels.

In general, the answer to the question of which is the best algorithm depends on the goal that has been set for defining if the dehazing procedure has worked effectively; if this goal is to produce images that are the most similar to the haze-free scene, then DehazeNet and Meng would be the best options (although the e metric values for Meng are very high when the haze is dense). If the goal is to obtain better contrast and more visibility of the objects present in the scene, then Berman and DCP perform better than the rest.

### 3.2. Subjective Evaluation

As we explained in Section 2.4, the survey has tackled two main issues: similarity between the haze-free and dehazed images, and the increase in visibility for the objects present in the scene after dehazing regardless of whether the restored image looks similar to the original haze-free image or not.

Regarding the first issue, the survey participants were asked to assign a rank from 5 to 1 according to the similarity between the dehazed and haze-free original images, with 5 being the most similar and 1 the least similar.

**Figure 8:**
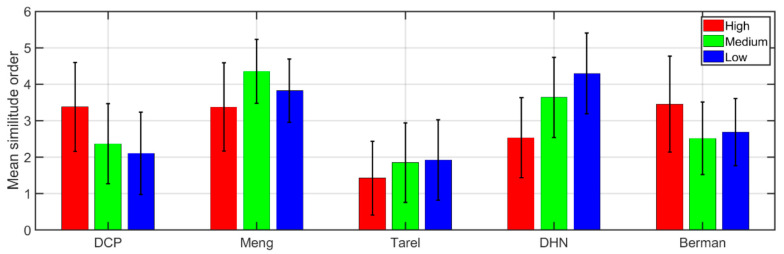
Average ranking (with standard deviation shown as error bars) of each algorithm across three wavelengths (530 nm, 620 nm and 710 nm) for the low and medium haze conditions, and two wavelengths (620 nm and 710 nm) for the high haze condition.

In Figure 8, the average rank value across participants and its standard deviation is shown for the three haze conditions and three representative wavelengths (530, 620 and 710 nm), except for the highest haze condition, for which only two wavelengths are averaged (620 and 710 nm), due to the limitations in image quality of the database used. A higher value will then indicate that the observers find the dehazed image more similar to the haze-free image.

In the high haze condition, DCP, Berman and Meng produced the most similar images to the haze-free reference, possibly because they are the only algorithms that are able to sufficiently dehaze the images to make some of the objects present start to be discernible. For the intermediate and low haze conditions, Meng and DehazeNet produced images that were more similar to the haze-free original, but Meng produced the highest rank values for medium haze, whilst DehazeNet had better ranking for low haze conditions. It is important to notice that DCP and Berman are among the least similar for medium and low haze conditions, possibly because the artificial increase in contrast that these algorithms produce creates a very noticeable difference in appearance between the dehazed and the original haze-free image. Tarel’s dehazed image was consistently ranked as the least similar, very likely because this algorithm was not working properly in the conditions tested (indoor scenes containing wide white areas).

**Figure 9:**
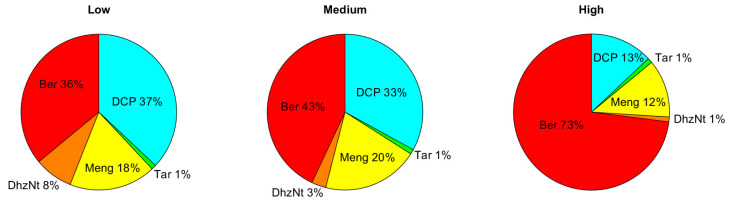
Pie chart with the preference results for each algorithm in each haze condition.

If the observers are asked to judge which algorithm has worked more effectively in achieving an increase in visibility of the objects in the scene, then the results are, as expected, different. In Figure 9, the percentage of preference obtained for each algorithm (percentage of observers who ranked the dehazed image produced by that algorithm as the one with the highest visibility) is shown for each haze condition. This percentage was obtained by pooling together the preference data for different wavelengths within the same haze condition.

For the highest haze level, the majority of observers chose Berman as the algorithm that improved visibility to a higher extent, followed by DCP and Meng. As the haze level decreased, so did the preference rate for Berman, whilst DCP was preferred by increasingly more participants. Meng was the third most preferred algorithm, achieving its best result for the intermediate haze level, whilst DehazeNet achieved better visibility (although still quite low in preference rate) for the lowest haze condition.

In general, the results of the survey are quite clear and easy to interpret. They do not always correlate directly with any of the metrics analyzed. Note, for instance, that the SSIM or PSNR results show Berman and DCP as the most different from the haze-free images for the highest haze condition (see Figure 7), whilst the observers’ answers to question 1 of the survey (see Figure 8) indicate that DCP and Berman are more similar to the haze-free image. Tarel was perceived by the observers as producing images that were not very similar to the haze free image, but its PSNR and SSIM results are not among the lowest. This result can be explained in terms of how the metrics work by simply assessing image intensity distributions without being able to discern an ensemble of attributes as the visual system does.

**Figure 10:**
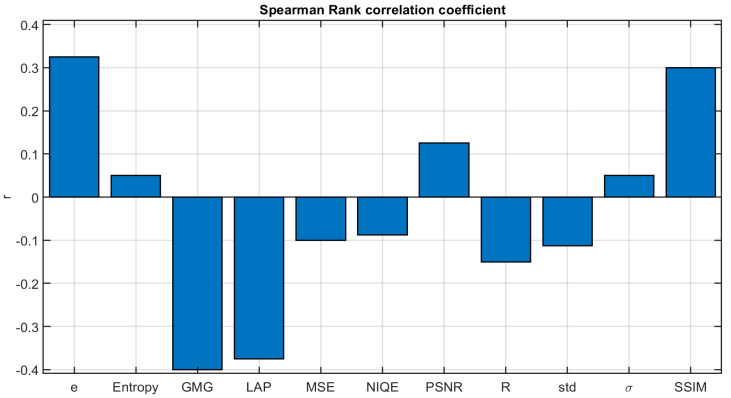
Average rank order correlation coefficient between observers’ answers to question 1 of the survey and the different metrics analyzed.

We have further analyzed this issue by computing the Spearman rank order correlation [36,37] for the eight conditions presented to the observers in the first question of the survey, comparing the observers’ individual ranks with the metric rank and averaging the rank order correlation values for the eight conditions and across observers. In Figure 10, we show the results for the eleven different metrics analyzed. This analysis shows that the correlation is in general low for all the metrics, with maximum values around 0.3–0.4. The correlation could also be negative if the metric had higher values for the most different images from the haze-free image, as is the case for LAP, GMG, MSE, NIQE, R and Std. These results have been used as one of the criteria for selecting the six most relevant metrics for analysis, as we explained in Section 2.3.

We have also evaluated the inter-observer variability by computing the rank order correlation coefficient using the observers’ rankings (all possible combinations taken two by two for each of the eight conditions shown in the survey). The average rank order correlation of the observers is in the range 0.38–0.54, with an average across conditions of 0.43. This suggests that the rank order correlation achieved by some of the metrics is close to the degree of agreement shown by the set of observers as a whole in their answers to question 1 of the survey. This relatively low correlation amongst observers is not unexpected if we consider the difficulty of ranking five different black and white images (some of them looking quite similar and, in some conditions, not very sharp either).

Nevertheless, there is some degree of agreement between the observer’s preferred algorithm according to the answers for question 2 and some of the metric results. Berman and DCP have consistently higher Std values for the three haze conditions, and they are also the two most preferred algorithms when the visibility of the objects in the scene is the criterion for preference. This might indicate that image contrast mediates the observers’ preference ranking in terms of visibility of the image. DehazeNet also presents good results, especially for low and medium haze levels in PSNR and SSIM metrics (related to similarity), and it is also among the best ranked according to question 1 (similarity between dehazed and haze-free images). Meng produces quite good results in similarity metrics, in agreement with the observers’ answers to question 1.

**Figure 11:**
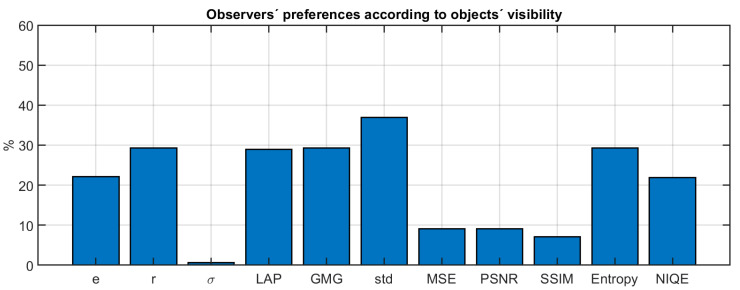
Percentage of agreement between the observers’ answers to question 2 of the survey and the different metrics analyzed.

We have further analyzed this issue by computing the percentage of observers which agreed with a given metric regarding the preferred dehazing result (according to question 2 of our survey). The results are shown in Figure 11.

Although the maximum values in Figure 11 are around 40% for the Std metric, this is not an unexpected result given the variety of backgrounds of the observers who participated in the survey and the high number of participants. If we consider the results of Figure 9, pooling together the three haze conditions, we obtain the result that 48% of the observers agreed on the selection of Berman as the preferred algorithm. The results for the Std metric are then quite close to the limit posed by the inter-observer variability. Some of the selected metrics tend to agree with the observers’ preference (such as Std, GMG and entropy). The metrics which judge quality according to the similarity with the haze-free image (MSE, PSNR and SSIM) show the least agreement with the observers, whilst the metrics which tend to judge quality in terms of “naturalness” (such as NIQE) are around 20% in agreement with our observer pool. This supports the hypothesis that the observers’ preference is not driven by similarity to the haze-free image or naturalness, but rather by the increase in contrast and detail enhancement that the dehazing algorithms are able to produce.

**Figure 12:**
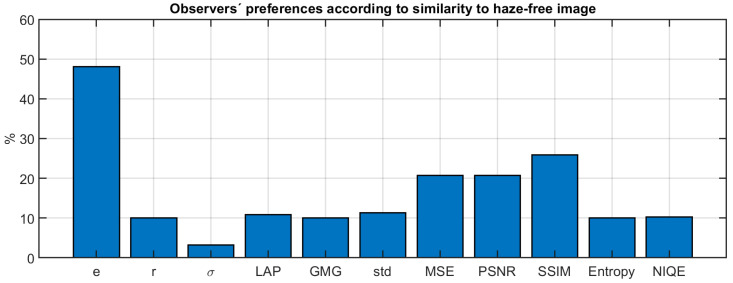
Percentage of agreement between the algorithm selected as best according to the observers’ answers to question 1 of the survey and the different metrics analyzed.

We have further tested this hypothesis by using the results to question 1 of the survey to extract the preferred algorithm (the one which was ranked with the highest vote) according to similarity with the haze-free image. Then, we re-computed the percentage of observers which are in agreement with a given metric’s preferred algorithm (see Figure 12).

We can see how now the similarity-based metrics (MSE, PSNR, SSIM) obtain higher levels of agreement with the observers than all others, except the e metric, which obtains even higher values than those shown in Figure 11. The level of agreement is in general lower than the one shown in Figure 11, which is expected because the rank order task was of higher complexity than the preference task. The results of this figure show that the observers were effectively selecting the best algorithm according to different criteria in question 1 and question 2 of the survey, as they were instructed to do.

## 4. Conclusions

In this study, we have analyzed the influence of two main factors in five state-of-the-art dehazing algorithms’ performance using a database of hazy spectral images: wavelength (within the visible range) and haze/fog level. We have also performed an objective analysis using eleven metrics commonly used in the literature, and a subjective analysis based on the judgements of 126 observers.

Regarding the objective analysis results, our results show that the quality of the dehazed images increases for longer wavelengths in most cases. This can be explained considering that the quality of the spectral images is higher in this range (due to the limitations of the capture imaging device) and, especially for low haze levels, the higher the wavelength is, the lower the attenuation present in the image. Regarding the influence of the haze/fog level, we have found a general trend towards decreasing quality in the majority of metrics for high haze levels, as expected.

The objective analysis allows us to see that the preferred algorithm would vary according to the selected metric. This makes sense, because the design strategies of the metrics are markedly different. For instance, a similarity metric would yield a low-quality value for an algorithm that causes a marked increment in contrast. In general, the dehazing algorithms tend to introduce some artifacts in the dehazed images, such as contrast boosting or spurious edges. The algorithms based on learning, such as DehazeNet, tend to introduce fewer artifacts, but their results might depend on the characteristics of the images used for training.

The results of the subjective analysis have yielded two main conclusions: the algorithm preferred by most observers (without taking into account the similarity to the haze-free image) is Berman, and the least effective is Tarel. This is somewhat surprising because Berman relies strongly on color in its underlying idea. When we asked the observers to rank the algorithms according to similarity with the haze-free image, DehazeNet and Meng obtained slightly better average ranking. The ranking task is more difficult to perform than the preference task, so the agreement between observers is also lower.

When we tried to analyze the correlation between the metrics and the observers’ results in the ranking task, we found that the e, GMG, LAP and SSIM metrics obtained a higher correlation with the observers, with similar values to the inter-observer’s correlation. Regarding the preference task, the metrics with the highest agreement with the observers are Std, GMG, LAP and entropy.

These results suggest that it is important to select metrics that are designed to work in agreement with the intended purpose of the dehazing algorithms that are to be evaluated. For instance, if one wants to dehaze images for better performance of a text recognition task, then contrast enhancement can be quite critical for achieving good results after dehazing. It would make sense to select metrics like GMG, Std or entropy for objective evaluation of the dehazing in this case (apart from the text recognition accuracy). If one wants to perform dehazing to include the images in a database along with haze-free scenes, then the similarity metrics (PSNR, SSIM) would work better to select the best dehazing algorithm.

In future studies, we plan to tackle an additional psychophysical experiment in which only special observers are considered, for example, flight pilots or car drivers, using images related to their jobs. We plan also to include other dehazing methods as well as extend the analysis to the infrared region of the spectrum. In addition, we want to check what is the best way for existing color dehazing methods to deal with spectral images: to duplicate the information at the three RGB channels, to restrict the methods to only one channel or to use different spectral bands at each RGB channel.

## Figures and Tables

**Table 1:** Summary of metric classification criteria.

Metric	Reference	Design Strategy	Selected for Section 3.1
e	Reduced	New edges	Yes
r	Reduced	New edges	No
σ	Full	Lost pixels	No
LAP	Non	Derivatives	No
GMG	Non	Derivatives	Yes
Std	Non	Contrast	Yes
MSE	Full	Similarity	No
PSNR	Full	Similarity	Yes
Entropy	Non	Information	Yes
SSIM	Full	Perceptual based	Yes
NIQE	Non	Perceptual based	No
